# Effects of dynamic individualized PEEP guided by driving pressure in laparoscopic surgery on postoperative atelectasis in elderly patients: a prospective randomized controlled trial

**DOI:** 10.1186/s12871-022-01613-9

**Published:** 2022-03-16

**Authors:** Qi Xu, Xiao Guo, Jiang liu, Si-xun Li, Hai-rui Ma, Fei-xiang Wang, Jing-yan Lin

**Affiliations:** 1 Department of Anesthesiology, Affiliated Hospital of North Sichuan Medical College, Nanchong, 637000 Sichuan China; 2 Department of Anesthesiology, North Sichuan Medical College, Nanchong, 637000 Sichuan China

**Keywords:** Driving pressure, Positive end-expiratory pressure, Atelectasis, Lung ultrasound, Laparoscopic surgery

## Abstract

**Background:**

Driving pressure (ΔP = Plateau pressure-PEEP) is highly correlated with postoperative pulmonary complications (PPCs) and appears to be a promising indicator for optimizing ventilator settings. We hypothesized that dynamic, individualized positive end-expiratory pressure (PEEP) guided by ΔP could reduce postoperative atelectasis and improve intraoperative oxygenation, respiratory mechanics, and reduce the incidence of PPCs on elderly patients undergoing laparoscopic surgery.

**Methods:**

Fifty-one elderly patients who were subject to laparoscopic surgery participated in this randomized trial. In the PEEP titration group (DV group), the PEEP titration was decremented to the lowest ΔP and repeated every 1 h. Additional procedures were also performed when performing predefined events that may be associated with lung collapse. In the constant PEEP group (PV group), a PEEP of 6 cmH_2_O was used throughout the surgery. Moreover, zero PEEP was applied during the entire procedure in the conventional ventilation group (CV group). The primary objective of this study was lung ultrasound score noted at the end of surgery and 15 min after admission to the post-anesthesia care unit (PACU) at 12 lung areas bilaterally. The secondary endpoints were perioperative oxygenation function, expiratory mechanics, and the incidence of the PPCs.

**Results:**

The lung ultrasound scores of the DV group were significantly lower than those in the PV group and CV group (*P* < 0.05), whereas there was no significant difference between the PV group and CV group (*P* > 0.05). The lung static compliance (Cstat) and ΔP at all the intraoperative time points in the DV group were significantly better compared to the PV group and the CV group (*p* < 0.05).

**Conclusions:**

Intraoperative titrated PEEP reduced postoperative lung atelectasis and improved respiratory mechanics in elderly patients undergoing laparoscopic surgery. Meanwhile, standard PEEP strategy is not superior to conventional ventilation in reducing postoperative pulmonary atelectasis in laparoscopic surgery.

## Introduction

In recent years, due to the advantages of a small incision and enhanced recovery after surgery, laparoscopic surgery has gradually become the primary abdominal surgery [1]. However, the establishment of pneumoperitoneum leads to the displacement of the diaphragm to the head, reduces the functional residual capacity (FRC), and promotes the formation of atelectasis, thus leading to impaired respiratory mechanics and gas exchange [2, 3]. More importantly, pulmonary atelectasis underlies the pathophysiology of PPCs, the development of which may prolong hospital stays and increase mortality in surgical patients [2–5]. Intraoperative lung protective ventilation strategy, which includes the combination of low tidal volume and adequate PEEP levels during operation, has been reported to improve respiratory mechanics and reduce the incidence and severity of atelectasis [6, 7].

Nevertheless, the setting of PEEP levels is controversial, especially in laparoscopic procedures. A previous study found that the optimal PEEP requirements of patients receiving protective ventilation during abdominal surgery anesthesia varied considerably [6]. Besides, individualized PEEP has advantages over fixed PEEP in improved respiratory mechanics and reduced incidence and severity of pulmonary atelectasis [6, 8]. Therefore, among lung-protective ventilation strategies, individualized PEEP is an important measure to prevent progressive alveolar collapse. A meta-analysis showed that postoperative pulmonary complications were associated with ΔP but not with tidal volume. In its mediation analysis, ΔP is the only important mediator of protective ventilation on pulmonary complications [9]. In addition, Park et al. [10] found that the application of ΔP in single-lung ventilation reduced postoperative pulmonary complications compared to conventional protected ventilation in thoracic surgery, and Gouri Mini et al. [11] found that individualized PEEP with ΔP titration reduced postoperative pulmonary atelectasis in open surgery. Therefore titrating PEEP to obtain the lowest ΔP may be an effective strategy to reduce the occurrence of atelectasis. However, the use of ΔP in laparoscopic surgery is limited by the formation of a pneumoperitoneum during laparoscopy, affecting chest wall compliance. Besides, PEEP was performed only once for individualized optimization in most studies, and the procedure was dynamic. Few studies have taken into account the effects of timing and manipulation on alveolar collapse, and the effect of dynamic, individualized PEEP guided by driving pressure on postoperative pulmonary atelectasis still requires many prospective studies.

Impairment of gas exchange associated with anesthesia is exacerbated with increasing age, making the elderly more susceptible to postoperative pulmonary complications [12]. The evidence for optimal PEEP during mechanical ventilation in elderly patients undergoing laparoscopic surgery is insufficient. Thus, we applied dynamic, individualized PEEP guided by ΔP in elderly patients undergoing laparoscopic surgery. We hypothesized that the individualized PEEP guided by ΔP could improve early postoperative atelectasis, intraoperative oxygenation function, pulmonary mechanics and reduce the incidence of PPCs.

## Materials and methods

We performed a prospective, double-blinded, randomized controlled trial at Affiliated Hospital of North Sichuan Medical College from January 2021 to July 2021. The trial protocol was approved by the Medical Ethics Committee of Affiliated Hospital of North Sichuan Medical College (2020ER180-1). The protocol was also registered in the Chinese Clinical Trials Registry https://www.chictr.org.cn/usercenter.aspx (ID: ChiCTR2100042568) on 23/01/2021. Written informed consent was obtained from all patients prior to inclusion.

### Participants

Inclusion criteria included patients older than 65 who were scheduled to undergo elective laparoscopic surgery of expected duration greater than 2 h; patients classified as American Society of Anesthesiologists (ASA) physical status II-III with a body mass index (BMI) less than 30 kg/m^2^. Patients will be excluded if they meet at least one of the following criteria: refusal to participate in the study, history of severe chronic obstructive pulmonary disease (COPD, GOLD III or IV), history of severe or uncontrolled bronchial asthma, history of severe restrictive lung disease, history of pulmonary metastases, history of any thoracic surgery, need for chest drainage prior to surgery, preoperative renal replacement therapy, congestive heart failure (NYHA: Class III or IV class).

A pilot study was performed on 17 patients to measure the lung ultrasound score at the end of the surgery to estimate the sample size. Sample size calculations were performed using PASS 15.0. The means and standard deviations for the DV group, PV group, and CV group were 10 ± 2.08, 11.80 ± 1.10, and 12.80 ± 2.49, respectively. Sample size calculations showed that 14 subjects per group were required to achieve 90% power with a Type I error of 0.05. A total of 51 patients (17 patients per group) were included in this trial considering an 80% adherence rate.

### Randomization and blinding technique

All enrolled patients were equally divided into three groups and administered with lung-protective ventilation with individualized PEEP guided by ΔP (DV group), lung-protective ventilation with standardized PEEP 6 cmH_2_O (PV group), and the conventional ventilation without PEEP (CV group), respectively. Randomization was done on the day before surgery using a web-based random-number generator (available at www.random.org) with no block size and stratification factors. The intervention protocol was kept in a closed, nontransparent, numbered envelope. An anesthetist who was not involved in designing the protocol for the study opened the envelope and set up the ventilator as specified in the envelope, and collected data throughout the procedure. The ventilation protocol blinded the patients and the researchers who performed the lung ultrasound and collected data on postoperative outcomes.

### Standard procedure

All patients refrained from eating or drinking for 8 h before surgery. Blood gas samples were collected through the radial artery catheter. All recruits were pre-oxygenated for 3 min. Sufentanil 0.3 µg/kg, cis-atracurium 0.10 mg/kg, and propofol 1.5 mg/kg were then injected intravenously for induction of anesthesia. Anesthesia was maintained using sevoflurane and intermittent administration of sufentanil and cis-atracurium. The anesthetic depth was titrated in all groups to maintain a bispectral index (BIS) range between 40 and 60. Hypotension was defined as the systolic pressure below 90 mmHg or 20% of the preoperative level, and vasoactive drugs were given for treatment.

### Ventilation protocol

Mechanical ventilation protocol was performed on the anesthesia machine (Datex-Ohmeda Aelite NXT). All groups were ventilated in volume-controlled mechanical ventilation mode with an inspiratory to expiratory ratio of 1:2 FiO_2_ was maintained at 1.0 during the induction period until extubation. The respiratory rate was started at 12 breaths/min and then adjusted to keep the end-expiratory carbon dioxide (P_ET_CO_2_) in the normal range of 35–40 mmHg. The patient’s ideal body weight (IBW) was predefined according to these formulas [13]: 45.5 + 0.91 × [height(cm)-152.4] for women or 50 + 0.91 × [height(cm)-152.4] for men. In the CV group, the tidal volume was set at 10 ml/kg IBW without PEEP or recruitment maneuvers (RM). In the PV group, patients were provided with a tidal volume of 8 ml/kg IBW and an intraoperative 6 cmH_2_O PEEP. In the DV group, based on a previous study [14], PEEP was increased from 5 cm H_2_O to 15 cmH_2_O before titration, with 5 cmH_2_O intervals for recruitment. Each PEEP level was maintained for 4–5 respiratory cycles (< 90 s required). During recruitment, the respiratory rate was 10 breaths/min, inspiratory: expiratory duration = 1:1, 30% inspiratory pause, and VT 8 ml/kg IBW. Recruitment was stopped if the plateau pressure (Pplat) reached 30 cmH_2_O. The second step was PEEP titration. PEEP was started at 10cmH_2_O and then reduced in 1 cmH_2_O interval to 4 cmH_2_O. After 10 breath cycles were maintained, ΔP was measured at each PEEP level. The PEEP indicating the lowest ΔP was selected. If multiple levels of PEEP showed the same lowest ΔP, the lowest PEEP was selected. The PEEP was titrated with a respiratory rate of 12 breaths/min, inspiratory: expiratory duration = 1: 2, 30% inspiratory interval, and VT 8 ml/kg IBW. And the same procedure was repeated every 1 h. Additional titration will be performed when performing predefined events that may be associated with lung collapse (application of surgical retractors, pneumoperitoneum inflation/deflation, tracheal tube disconnection, tracheal suctioning, Trendelenburg position). And we will wait until the patient is in a stable state (e.g., after completing the position change) to re-titrate the optimal PEEP. Before each PEEP adjustment, muscle relaxation and hemodynamic status stability were ensured. All interventions were performed immediately after tracheal intubation.

### Data source and collection

**Fig. 1:**
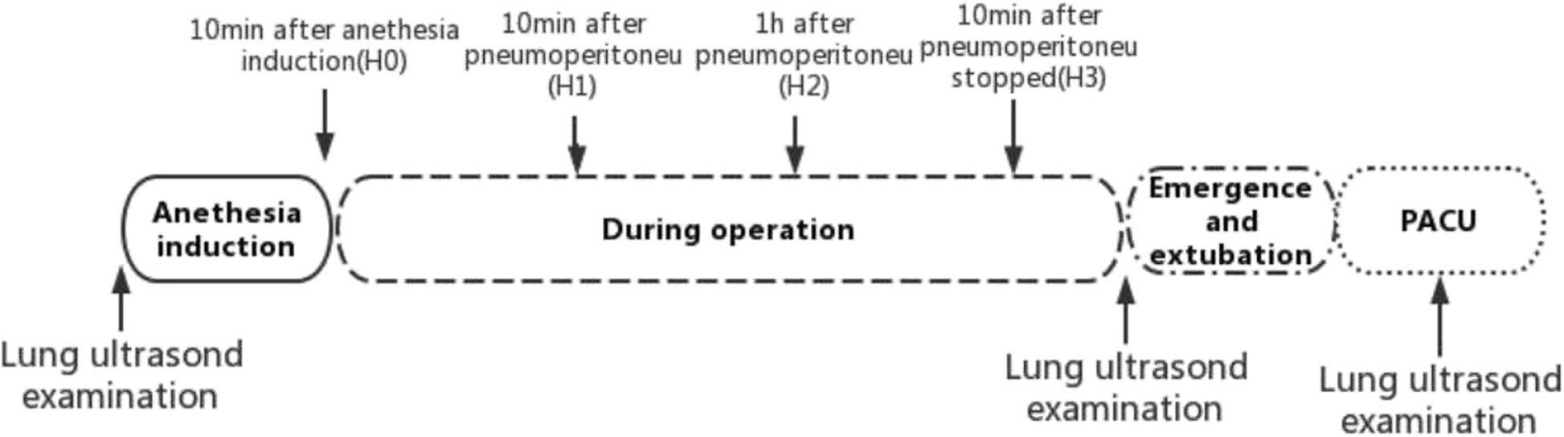
Study protocol. Four predefined time points (H0–H3) when intraoperative ventilatory parameters were recorded and arterial blood gas analysis was performed and three-time points of ultrasound examination

Demographic characteristics were recorded including, age, sex, BMI, ASA physical status, coexisting medical conditions, and smoking history. The volume of intravenous fluid, the volume of blood loss, urine output, and respiratory risk in surgical patients in Catalonia (ARISCAT) score [15] were recorded. Arterial blood gas samples were collected for analysis at H0 (10 min after tracheal intubation), H1 (10 min after pneumoperitoneum establishment), H2 (1 h after pneumoperitoneum establishment) and H3 (10 min after pneumoperitoneum cessation), respectively. The ΔP was calculated following the predefined formula as Pplat – PEEP. The Cstat was calculated following the predefined formula as Vt/(Pplat – PEEP), with the Pplat being measured during the standard ventilation setting using an inspiratory pause at 20% of the inspiratory time. The ΔP and Cstat were recorded at H0, H1, H2, and H3, respectively. Lung ultrasound was performed before surgery (T0), at the end of surgery but before extubation (T1), and 15 min after PACU admission (T2), respectively (Fig. 1). The occurrence of PPCs [16] (more than 3 of the following 6 new conditions: cough, increased sputum, dyspnea, chest pain, temperature above 38 °C, HR > 100 beats/min) within 2 and 7 days postoperatively was recorded.

### Primary and secondary endpoints

**Fig. 2:**
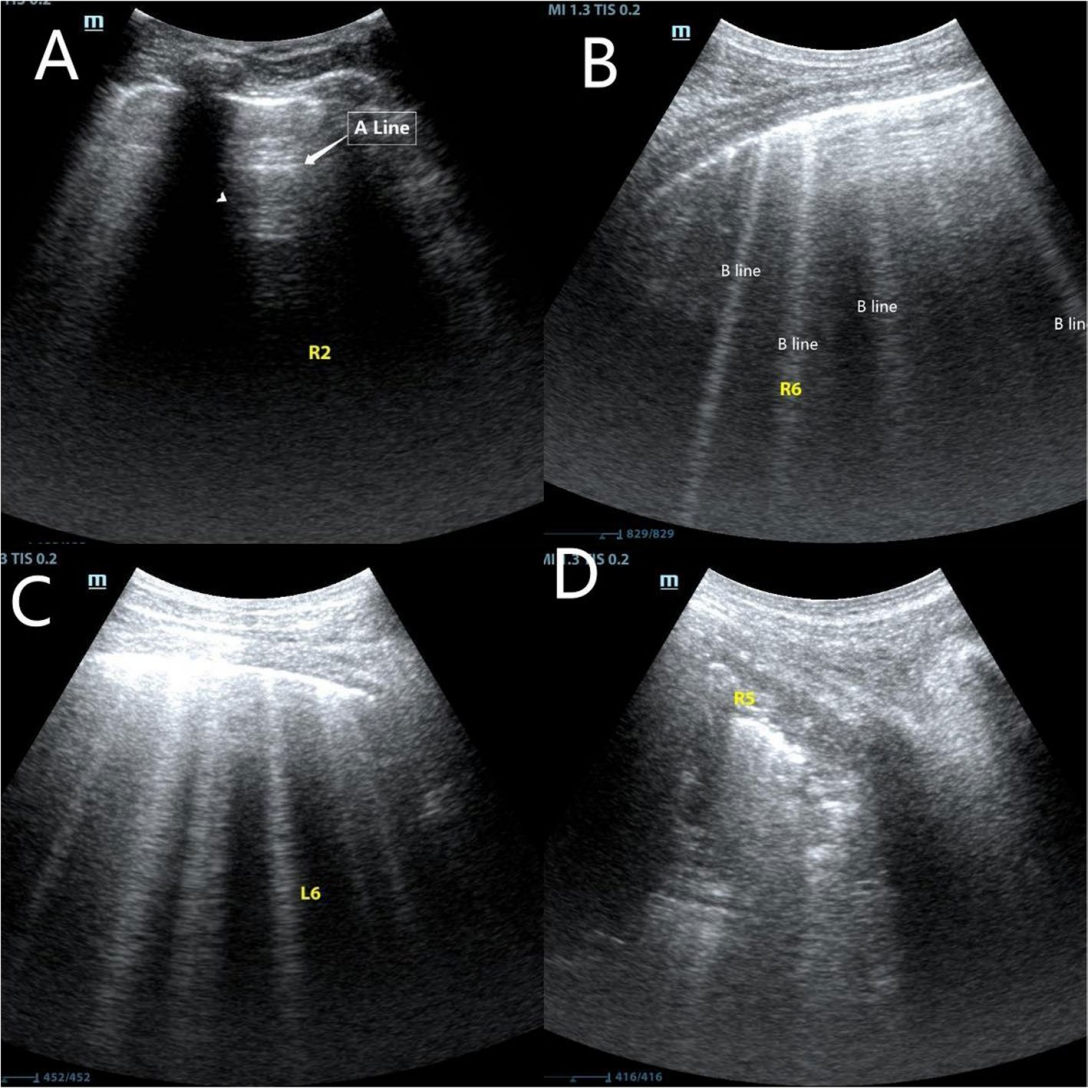
Lung ultrasound scores determined by the number of B lines and subpleural consolidation. **A** Normal aeration with 0–2 B lines, score = 0, **B** a small loss of aeration with ≥ 3 B lines, score = 1, **C** a moderate loss of aeration with multiple coalescent B lines or small subpleural consolidation, score = 2, and **D** a severe loss of aeration with consolidation or large subpleural consolidation, score = 3. (The yellow letters in the picture are the markings made in the trial. R and L represent the right and left hemithorax, and the numbers represent the subdivisions.)

The primary endpoint was the absolute difference in the three lung ultrasound scores measured at T0-T2. By using ultrasound (Mindray, M9), atelectasis was examined by a trained investigator blinded to the group allocation. Ultrasound examination was conducted at three predefined time points mentioned above. Based on a previous study [17, 18], the thorax was divided into 12 segments. The lung ultrasound score is 0 to 3, based on the B-line count and the degree of subpleural solidity. A total score of 0–36 was obtained by summing the scores of the 12 segments (Fig. 2). The secondary endpoints were the differences among the three groups regarding Partial pressure of arterial oxygen (PaO_2_), Alveolar-arterial oxygen partial pressure difference (A-aDO_2_)_,_ intraoperative Cstat, driving pressure, and the occurrence of PPCs within 2 and 7 days postoperatively.

### Statistics

All analyses were performed using IBM SPSS Statistics 25.0. the Kolmogorov–Smirnov test was used to test for a normal distribution. Normally distributed data were reported as mean ± [standard deviation (SD)] and analyzed using one-way analysis of variance (ANOVA) or repeated-measures ANOVA. Non-normally distributed data were analyzed using the Kruskal–Wallis test, and between-group tests were analyzed using the Kruskal–Wallis one-way ANOVA. Categorical variables were compared using the chi-square test and the rank-sum test. Post hoc analyses were conducted using the Bonferroni correction method. Statistically significant was considered to be a *p*-value of less than 0.05.

## Results

**Fig.3:**
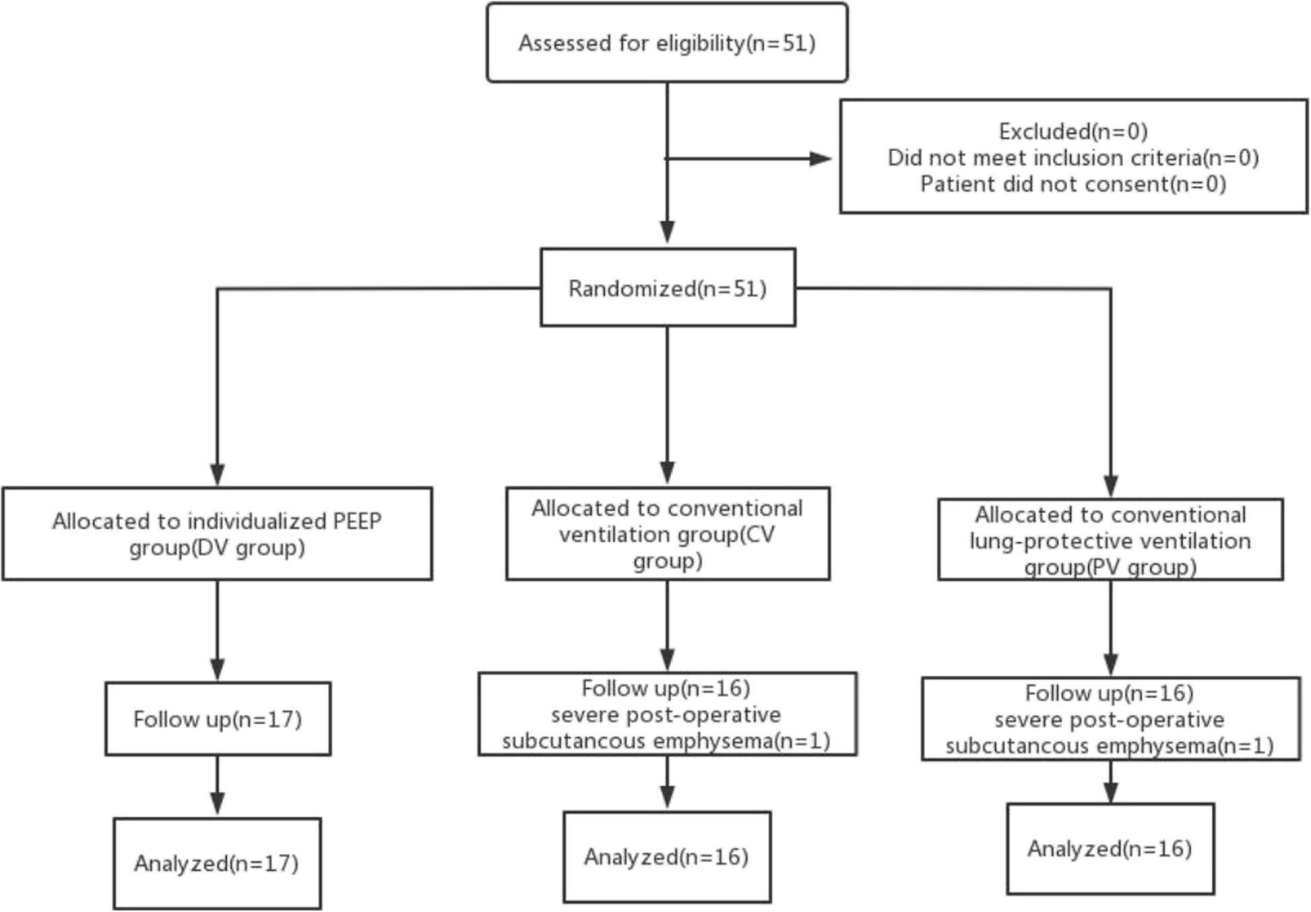
Flow diagram representing patient enrollment, group assignment, and analysis

Fifty-one patients were initially assessed for eligibility. Two patients due to severe postoperative subcutaneous emphysema were excluded from the study. Therefore, 49 patients were randomized into three groups. The registration flow chart is shown in Fig. 3. The demographic characteristics of the participants are shown in Table 1, and the surgical and anesthetic characteristics are shown in Table 2.

**Table1:** Patients characteristics among groups

	DV group(*n* = 17)	PV group(*n* = 16)	CV group(*n* = 16)	*P* value
Ages (years)	70(5)	68(4)	71(8)	0.221
Gender (male/female,n)	8/9	12/4	7/9	0.147
BMI (kg/m^2^)	21 ± 2	23 ± 3	22 ± 3	0.397
ASA Physical status (II/III, n)	14/3	15/1	13/3	0.532
Current smoker, n(%)	3(18)	1(6)	4(25)	0.351
History of diabetes mellitus, n(%)	1(6)	4(25)	3(19)	0.315
History of hypertension, n(%)	7(41)	6(38)	5(31)	0.837

Data are presented as mean ± standard deviation (SD), median and interquartile range (IQR), or proportion, as applicable

*ASA* American Society of Anesthesiologists, *BMI* Body Mass Index, *IQR* Interquartile Range

**Table2:** The surgical and anesthesiological characteristics among groups

	DV group(*n* = 17)	PV group(*n* = 16)	CV group(*n* = 16)	*P* value
Anesthesia time (min)	249 ± 65	269 ± 64	232 ± 58	0.247
Surgery time (min)	180(113)	203(108)	195(90)	0.590
Intravenous fluid volume(ml)	2415 ± 1097	2249 ± 509	2275 ± 454	0.838
Blood loss(ml)	100(150)	100(150)	50(118)	0.298
Urine output (ml)	350(470)	300(450)	450(450)	0.719
Induction time (min)	5(2)	5(2)	5(1)	0.517
Number of laryngoscopies	1(1)	1(1)	1(0)	0.341
Posture (Supine position/Lithotomy position, n)	5/12	8/8	7/9	0.465
Non-Trendelenburg position, n(%)	5(29)	7(44)	7(44)	0.618
ARISCAT score				0.175
< 26 points, n(%)	7(41)	5(31)	4(25)	
26–44 points, n(%)	9(53)	9(56)	10(63)	
> 44 points, n(%)	1(6)	2(13)	2(13)	
Use of vasopressor, n(%)	11(65)	13(81)	13(81)	0.552
Incidence of hypotension, n (%)	11(65)	13(81)	13(81)	0.552
Sufentanil(ug)	42.79 ± 11.92	43.94 ± 9.17	45.94 ± 9.79	0.682
Cis-atracurium(mg)	18.03 ± 6.13	17.13 ± 4.62	17.00 ± 5.16	0.833
Types of surgery				0.079
Gastrectomy, n(%)	5(29)	7(43)	8(50)	
Colectomy, n(%)	2(12)	2(13)	1(6)	
Radical resection of rectal carcinoma, n(%)	10(59)	7(44)	7(44)	

Data are presented as mean ± standard deviation (SD), median and interquartile range (IQR), or proportion, as applicable

### Primary outcome

**Fig. 4:**
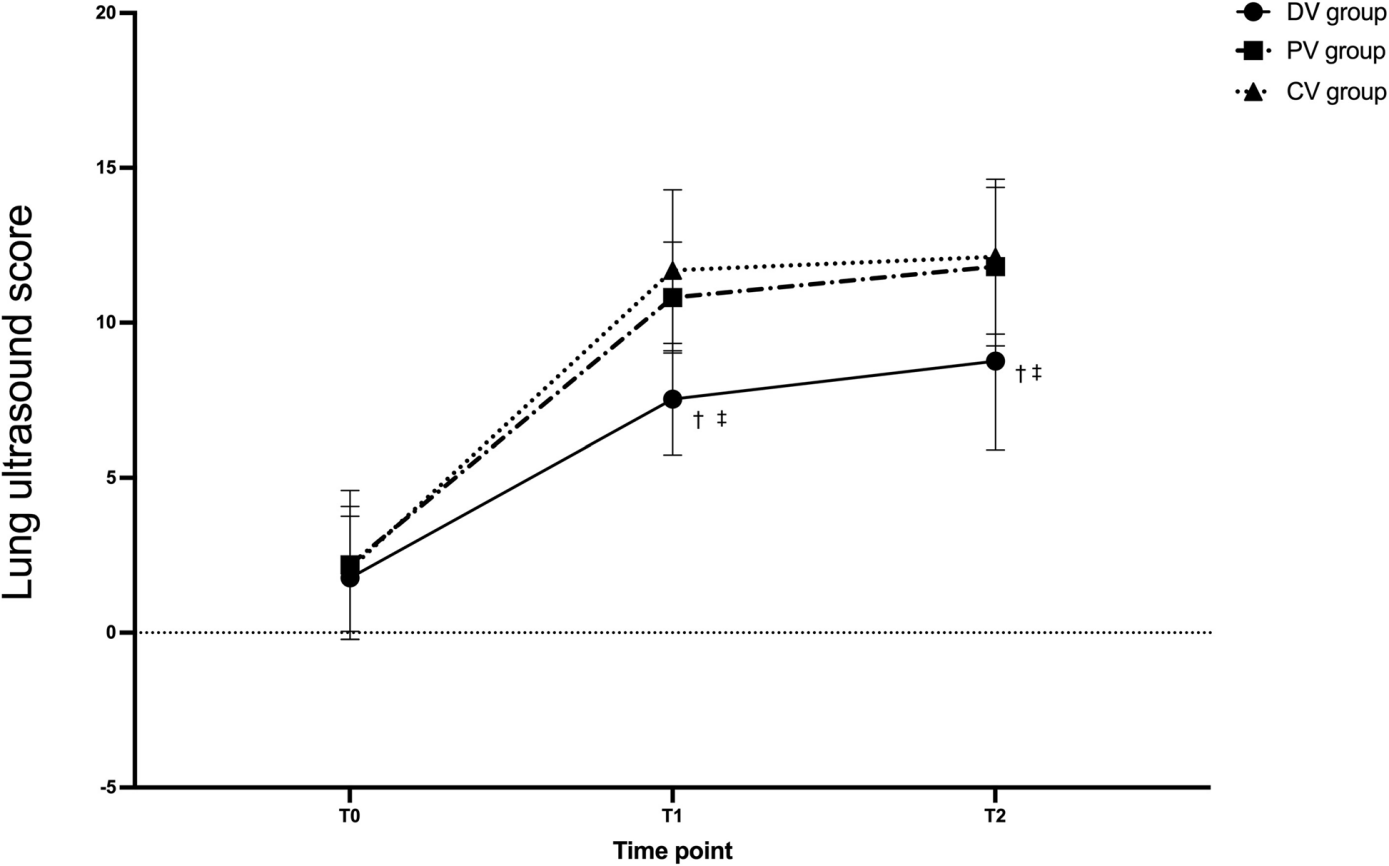
Lung ultrasound scores variations over time among three groups. Data are presented as mean ± SDs. T0: Before surgery; T1: At the end of the surgery but before extubation; T2: 15 min after the PACU admission; ^†^compared with the PV group, the difference was significant at 0.05 level; ^‡^compared with the CV group, the difference was significant at 0.05 level

Repeated-measures ANOVA results showed a time-group interaction for lung ultrasound scores. At T1 and T2, the lung ultrasound scores of the DV group were significantly lower than those in the PV group and CV group (*P* < 0.05), and there had no significant difference between the PV group and CV group (*P* > 0.05) (Fig. 4). The optimal PEEP in the individualized group was determined as the median (interquartile range). The optimal PEEP before pneumoperitoneum establishment is 5 (4–6) cmH_2_O, 10 min after pneumoperitoneum establishment is 6 (3–9) cmH_2_O, 1 h after pneumoperitoneum establishment is 7 (3–11) cmH_2_O and 10 min after the end of pneumoperitoneum is 4 (2–6) cmH_2_O (Table 3).

**Table3:** The optimal PEEP for titration at each time point (*n* = 17)

	H0	H1	H2	H3
PEEP (cmH_2_O)	5(1)	6(3)	7(4)	4(2)

Data are presented as median and interquartile range (IQR)

### Secondary outcomes

**Fig. 5:**
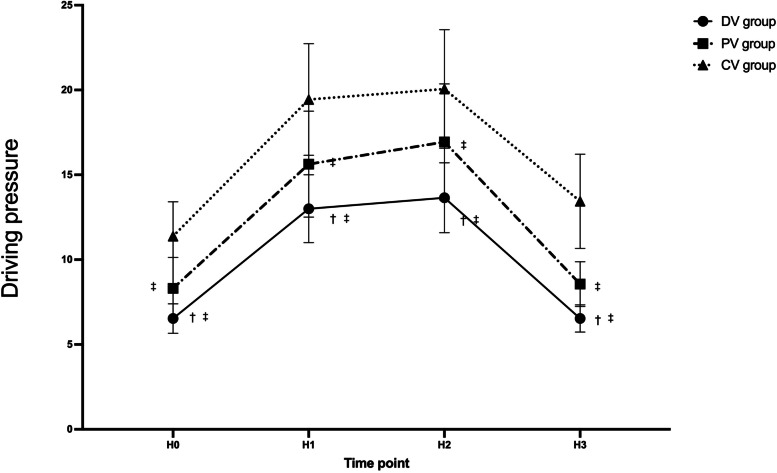
Driving pressure variations over time among three groups. Data are presented as mean ± SDs. H0: 10 min after endotracheal intubation; H1: 10 min after pneumoperitoneum; H2: 1 h after pneumoperitoneum; H3:10 min after pneumoperitoneum stopped. ^†^compared with the PV group, the difference was significant at 0.05 level. ^‡^compared with the CV group, the difference was significant at 0.05 level

Repeated measures ANOVA results showed a time-group interaction for ΔP and Cstat as well. At each time point, the ΔP was significantly lower in the PV and DV group compared to the CV group (*P* < 0.05), and it was significantly lower in the DV group than in the PV group (*P* < 0.05) (Fig. 5).

**Fig. 6:**
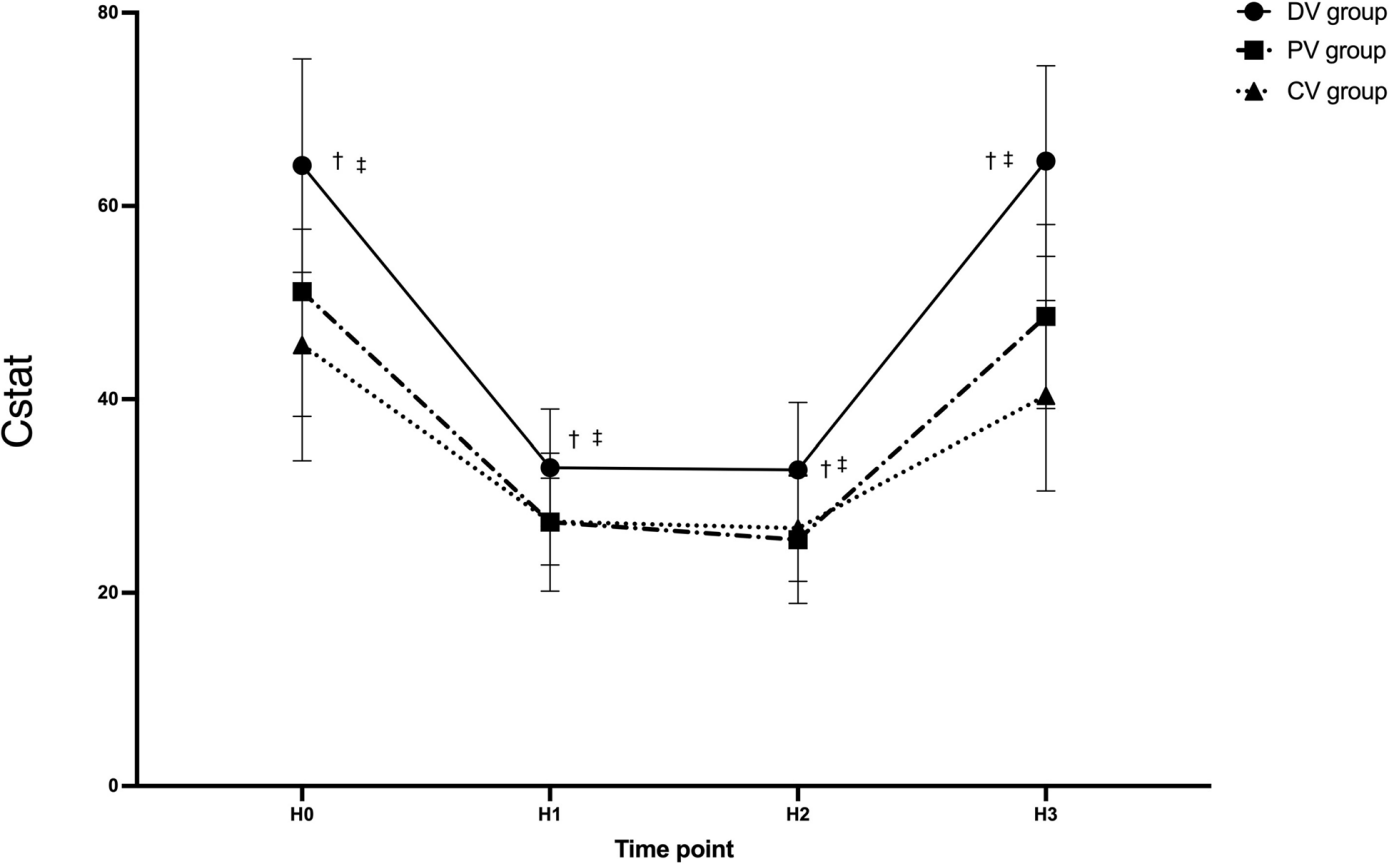
Cstat variations over time among three groups. Data are presented as mean ± SDs. H0: 10 min after endotracheal intubation; H1: 10 min after pneumoperitoneum; H2: 1 h after pneumoperitoneum; H3:10 min after pneumoperitoneu stopped. ^†^compared with the PV group, the difference was significant at 0.05 level. ^‡^compared with the CV group, the difference was significant at 0.05 level

As Cstat was considered, the PV group and the CV group had lower Cstat compared with the DV group at each time point (*P* < 0.05), there was no significant difference between the PV and CV group (*P* > 0.05) (Fig. 6).

**Fig. 7:**
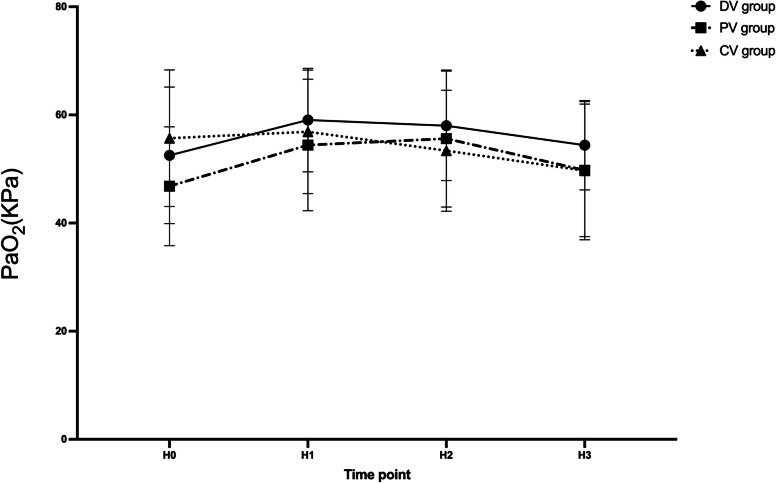
PaO_2_ variations over time among three groups. Data are presented as mean ± SDs. H0: 10 min after endotracheal intubation; H1: 10 min after pneumoperitoneum; H2: 1 h after pneumoperitoneum; H3:10 min after pneumoperitoneum stopped. ^†^compared with the PV group, the difference was significant at 0.05 level. ^‡^compared with the CV group, the difference was significant at 0.05 level

**Fig. 8:**
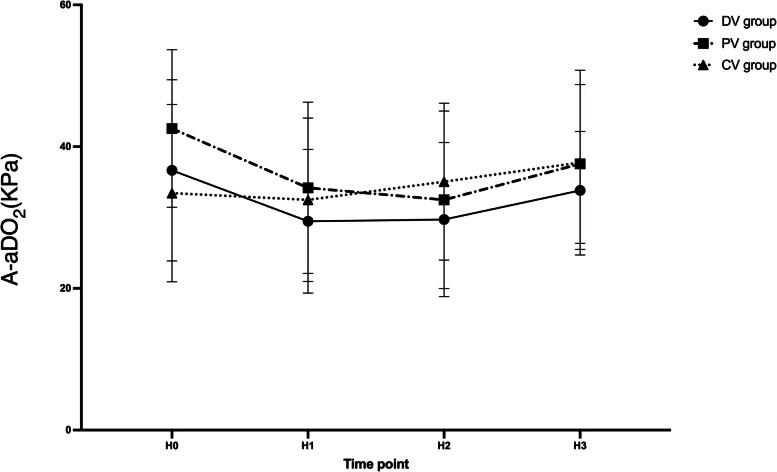
A-aDO_2_ variations over time among three groups. Data are presented as mean ± SDs. H0: 10 min after endotracheal intubation; H1: 10 min after pneumoperitoneum; H2: 1 h after pneumoperitoneum; H3:10 min after pneumoperitoneum stopped. ^†^compared with PV group the difference was significant at 0.05 level. ^‡^compared with CV group the difference was significant at 0.05 level

For PaO_2_ and A-aDO_2_, no time-group interaction effects were detected by repeated-measures ANOVA (*P* > 0.05). The PaO_2_ and A-aDO_2_ are shown in Fig. 7 and Fig. 8. There was no significant difference in the hospital stay or the occurrence of PPCs within 2 and 7 days postoperatively among the three groups of patients (*P* > 0.05). There was no statistical difference in the rate of vasoactive drug use and the incidence of hypotension among the three groups (*P* = 0.552).

## Discussion

This study examined the effect of dynamic, individualized PEEP guided by driving pressure in laparoscopic surgery on postoperative atelectasis in elderly patients. We have found that dynamic, individualized PEEP guided by ΔP decreases early postoperative pulmonary atelectasis. And the advantage of reducing pulmonary atelectasis did not disappear immediately after extubation. Those patients ventilated with dynamic, individualized PEEP reduce ΔP, and improve Cstat compared with a standard PEEP of 6 cmH_2_O and conventional ventilation. Meanwhile, standard PEEP strategy is not superior to conventional ventilation for reducing postoperative pulmonary atelectasis in elder patients undergoing laparoscopic surgery.

Driving pressure reflects global lung strain [19]. A meta-analysis [9] showed that postoperative pulmonary complications were associated with ΔP, but not with tidal volume. In its mediation analysis, ΔP is the only important mediator of protective ventilation on pulmonary complications. However, the use of ΔP to assess lung strain in laparoscopy is controversial. The ΔP measured during mechanical ventilation has two components, one is related to pulmonary expansion, and the other is related to chest wall expansion [19]. Therefore, the ΔP depends on the characteristics of the entire respiratory system, not just the lung characteristics, and may mislead the setting of mechanical ventilation when chest wall compliance is abnormal. Carbon dioxide pneumoperitoneum increases chest wall compliance but does not affect lung compliance. A higher ΔP during abdominal closure surgery is usually considered innocent. However, recent research negates this hypothesis [20]. This study point to a stronger correlation between ΔP in laparoscopic surgery than in abdominal surgery in the analysis of ΔP and postoperative pulmonary complications. The lung ultrasound results showed that the DV group had a mean reduction in lung ultrasound score minus 3.28 points relative to the PV group (*P* < 0.05) and a mean reduction of 4.16 points relative to the CV group (*P* < 0.05) at the end of surgery. The advantage of reducing pulmonary atelectasis did not disappear immediately after extubation (Fig. 4). Recent studies have shown that in patients with acute respiratory distress syndrome (ARDS), each point change in lung ultrasound score is equivalent to a 72 ml change in ventilatory function [21]. Although this result may not apply to our subjects, the reduction in lung ultrasound scores still suggests improved pulmonary ventilation in the individualized PEEP group. A previous study has concluded that the use of lung ultrasound in patients undergoing general anesthesia has a clinically meaningful mean difference of 4 points in lung ultrasound scores based on the clinical experience of the investigators, but there is insufficient evidence to support this. Therefore, the relationship between lung ultrasound scores and clinical outcomes remains to be further explored. However, there was no statistical difference in lung ultrasound scores between the PV and CV groups (Fig. 4). It is because the tidal volume was different between the two groups, and the tidal volume affects the magnitude of the driving pressure [22]. Cstat is tidal volume/driving pressure [14], and there was no difference in Cstat between the PV and CV groups (Fig. 6), resulting in no statistical difference in lung ultrasound scores. Our study applied ΔP to laparoscopic surgery and found that individualized PEEP guided by ΔP can reduce postoperative atelectasis. Besides, our results showed that individualized PEEP guided by ΔP reduces driving pressure at each time point (Fig. 5). This result means that the lung strain decreases, and there is a relative balance between atelectasis and hyper lung expansion.

One study found significant within-patient variability in individualized PEEP, ranging from 0 to 87% [8]. Even if the PEEP is optimized at the beginning of the procedure, continuous PEEP is not sufficient to maintain this optimization under the dynamic conditions of laparoscopic surgery. Besides, a previous study [23] compared protected ventilation with conventional ventilation, found that some subjects in the conventional ventilation group showed better lung ventilation than subjects in the protected ventilation group. This inter-patient variability also emphasizes the importance of individualized and dynamic monitoring of pulmonary ventilation. During laparoscopic surgery, procedures such as establishing a pneumoperitoneum, changing positions, and suctioning may lead to alveolar collapse. We titrated the optimal PEEP multiple times in this study, and we found differences in the median optimal PEEP for all four-time points. The optimal PEEP may differ even for the same individual with all conditions held constant. Our individualized PEEP titration strategy takes into account both individualized differences in respiratory compliance and the transformation of compliance over time and across manipulations. In addition, the titration process of individualized PEEP is simple and does not require additional instrumentation. Each titration can be done quickly, and even multiple titrations do not add significantly to the anesthesiologist’s workload.

Previous studies have found that maintaining PEEP above at least 10 cmH_2_O during laparoscopic surgery leads to more homogeneous ventilation and favorable physiological outcomes. However, a study found no difference in the postoperative lung function between the high PEEP and conventional PEEP groups but an increased need for vasopressors and fluids in the high PEEP group [24]. In addition, inflammatory markers were significantly increased in pigs exposed to high PEEP levels compared to the low PEEP level group after 8 h of unprocedural low volume ventilation, suggesting that high PEEP may cause lung injury [25]. Elderly patients are often associated with various complications, and it is unknown whether the advantages of reducing pulmonary atelectasis can outweigh its disadvantages. Previous studies have shown that carbon dioxide pneumoperitoneum reduces left ventricular preload [26], which is exacerbated by PEEP [27, 28]. Therefore, in our study, we titrated within 10 cmH_2_O for elderly patients, seeking to reduce pulmonary atelectasis while minimizing the deficits associated with high PEEP. Our study found that a moderate PEEP level within 10 cmH_2_O, with dynamic individualized titration, also reduced postoperative pulmonary atelectasis and improved respiratory mechanics. Significantly, individualized PEEP did not increase the use of vasopressors and fluids (Table. 2). Our study found no difference in perioperative oxygenation function among the three groups of subjects. But Davide D’Antini et al. [29] found that individualized PEEP improved oxygenation in patients undergoing laparoscopic cholecystectomy. It may be due to our intraoperative use of pure oxygen, narrowing the advantage of the improved oxygenation function due to optimized PEEP.

Although our study found that individualized PEEP guided by ΔP reduced postoperative pulmonary atelectasis and improved respiratory mechanics, it still did not influence the incidence of PPCs. Theoretically, atelectasis is associated with reduced pulmonary compliance and impaired oxygenation; the adverse effects persist into the postoperative period and prolong the patient’s hospital stay [30]. However, in an international expert panel-based consensus recommendation for lung-protective ventilation in surgical patients, it was found that the benefits of individualized PEEP to improve oxygen and end-expiratory lung volume (EELV) and respiratory mechanics during ventilation may disappear quickly after extubation [31]. Therefore, mechanical ventilation should be aimed at optimizing respiratory function and minimizing factors associated with perioperative complications. Additional studies are needed to quantify whether these positive intraoperative effects on the mechanical performance of ventilation have a clinically meaningful impact on prognosis.

Here are some limitations to our study. First, this is a single-centered trial. Second, the relatively small sample size is due to the fact that we calculated the sample size based on postoperative lung ultrasound scores as the primary index, and it was not adequately powered to detect a difference in PPCs. Third, studies have found that high FiO_2_ affects the area of pulmonary atelectasis. However, due to limited conditions, we had to use pure oxygen, which may lead to high lung ultrasound scores. And this condition may result in no statistical difference in terms of gas exchange. Although, recommendations and current evidence for optimal FiO_2_ use during intraoperative mechanical ventilation are not yet sufficient [31]. The effect of individualized PEEP guided by driving pressure on gas exchange still needs to be supported by more substantial evidence. Forth, we did not compare the differences between Trendelenburg and non-Trendelenburg. The effect of position change on postoperative pulmonary atelectasis need further investigation.

## Conclusion

The dynamic, individualized PEEP guided by driving pressure reduces early postoperative pulmonary atelectasis. The advantage of reducing pulmonary atelectasis did not disappear immediately after extubation. Also, respiratory mechanics improved in patients ventilated with dynamic, individualized PEEP in the perioperative period. Meanwhile, standard PEEP strategy is not superior to conventional ventilation in reducing postoperative pulmonary atelectasis in laparoscopic surgery.

## Data Availability

The datasets used during the current study are available from the corresponding author on reasonable request.

## Abbreviations

ΔP: Driving pressure
PPCs: Postoperative pulmonary complications
PEEP: Positive end-expiratory pressure
PACU: Post-anesthesia care unit
Cstat: Lung static compliance
FRC: Functional residual capacity
ASA: American society of anesthesiologists
COPD: Chronic obstructive pulmonary disease
NYHA: New York heart association
BIS: Bispectral index
PETCO_2_: End-tidal carbon dioxide
IBW: Ideal body weight
RM: Recruitment maneuvers
Pplat: Plateau pressure
PaO_2_: Partial pressure of arterial oxygen
A-aDO_2_: Alveolar-arterial oxygen partial pressure difference
ARISCAT: Respiratory risk in surgical patients in Catalonia
BMI: Body mass index
ANOVA: One-way analysis of variance
SD: Standard deviation
IQR: Interquartile range
ARDS: Acute respiratory distress syndrome
EELV: End-expiratory lung volume
